# Fear of Being Laughed at in Children and Adolescents: Exploring the Importance of Overweight, Underweight, and Teasing

**DOI:** 10.3389/fpsyg.2018.01447

**Published:** 2018-08-14

**Authors:** Carl-Walter Kohlmann, Heike Eschenbeck, Uwe Heim-Dreger, Michael Hock, Tracey Platt, Willibald Ruch

**Affiliations:** ^1^Psychology, University of Education Schwäbisch Gmünd, Schwäbisch Gmünd, Germany; ^2^Psychology, University of Bamberg, Bamberg, Germany; ^3^Institute of Sport and Human Sciences, University of Wolverhampton, Wolverhampton, United Kingdom; ^4^Department of Psychology, University of Zurich, Zurich, Switzerland

**Keywords:** gelotophobia, teasing, victimization, overweight, underweight, body image, well-being

## Abstract

Weight bias toward obese youths is often accompanied by the experience of psychological stress in those affected. Therefore, the fear of being laughed at (i.e., gelotophobia) in overweight children and adolescents can be rather serious. In four explorative studies, the importance of relative weight, self-awareness of weight (incl. satisfaction with weight), experiences of teasing and ridicule, as well as the role of social-evaluative situations in school were analyzed with regard to gelotophobia. In two online interviews of adults with pronounced gelotophobia (Study I: 102 English-speaking participants, Study II: 22 German-speaking participants) relating to reasons they assumed for their development of gelotophobia, there was evidence of injurious appearance-related experiences during childhood and adolescence. In Study III (75 Swiss adolescents) associations between the experience of weight-related teasing and mockery with overweight, self-perceptions of weight, and gelotophobia were analyzed. Especially in girls, overweight was associated with the experience of weight-related teasing and ridicule, which in turn was accompanied by gelotophobia. Study IV included 178 German adolescents who were asked to report their body image (“Do you think you are… too thin, just the right weight, or too fat?”). In addition, gelotophobia, teasing, BMI based on self-reports, and joy at school were measured. In particular, girls who felt too fat and boys who felt too thin reported teasing. Teasing was related to diminished joy at school and to gelotophobia. Among boys, underweight mediated by weight-related teasing contributed to gelotophobia. The results suggest that more research should be devoted to gelotophobia and the experience of weight-related teasing and mocking to better understand factors contributing to the well-being of children and adolescents with weight problems.

## Introduction

The aim of the present study is to explore the role of the fear of being laughed at (i.e., gelotophobia) in the context of overweight, teasing and well-being in children and adolescents.

Gelotophobia describes the fear of being an object of laughter (for an overview, see Ruch et al., 2014). Gelotophobia is conceptualized as an individual difference variable considerably varying in non-clinical samples (Ruch and Proyer, 2008). Individuals high in gelotophobia fear exposing themselves to others because they expect that others screen them for evidence of ridiculousness. Experiences of teasing and victimization (including being a target of destructive humor) during childhood and adolescence seem to play a role in the development of gelotophobia (Ruch et al., 2010).

Being teased about one’s weight is common among overweight and obese adolescents (Puhl and Latner, 2007; Haines et al., 2013). Obesity is associated with impaired well-being and mental health in children and adolescents (Eschenbeck et al., 2009; Griffiths et al., 2010). Weight bias toward obese youths seems to play a central role in the association between overweight and emotional well-being (Eisenberg et al., 2003). Weight-based teasing from peers and parents in adolescence can even result in weight gain, unhealthy weight control and eating to cope 15 years later (Puhl et al., 2017).

Studies have demonstrated that weight bias begins early in childhood. It becomes worse as children get older (Puhl and Latner, 2007). Puhl et al. (2008) applied an online interview with a sample of overweight and obese adults to identify and describe their subjective experiences of weight bias. When asked to describe their worst experiences of weight stigmatization participants reported experiencing weight stigma across a wide range of contexts and involving a variety of interpersonal sources. Most of the participants (77%) reported verbal bias, which included intentional negative comments, insults, derogatory names, teasing, ridicule or being made fun of because their weight. Emotional consequences of weight stigmatization were not assessed directly. However, when asked about what others should know about what it is like to be overweight besides mostly weight-based responses (e.g., difficulty of weight loss, physical challenges of excess weight) also emotional aspects have been reported (e.g., depressive feelings, feelings hurt, humiliation, embarrassment, sadness, and sorrow) in 36% of the interviews.

For individuals with pronounced gelotophobia, teasing experiences are documented as well. For example, adults high in gelotophobia reported that they have been teased quiet often during school time, that teachers made fun of them during lessons, and that they felt punished by their parents by means of ironic and sarcastic comments (Ruch et al., 2010). Edwards et al. (2010) found that among undergraduate students gelotophobia was positively correlated with a history of being teased. Teasing domains included social behavior, academic excellence, performance, and appearance. The appearance scale was comprised of several aspects of appearance (e.g., color or style of hair, wore glasses, weight, fatter than other kids). Therefore, the fear of being laughed at (i.e., gelotophobia) particularly in overweight children and adolescents can be rather serious. However, research combining the perspectives on teasing and victimization in overweight and obese children and adolescents on the one hand and teasing and victimization as related to gelotophobia on the other hand, has not yet received systematic attention.

To study the role of gelotophobia in relation to overweight, teasing, and emotional well-being in children and adolescents, two different perspectives were combined: do adults with gelotophobia remember weight-related teasing in younger age? Is weight-related teasing a mediator between weight status and gelotophobia in children and adolescents?

The following research questions will be addressed: (1) Are physical appearance and weight-related teasing and ridicule in childhood and adolescence among the assumed reasons for being laughed at in the memories of gelotophobic adults? (2) Is the experience of weight-based teasing a mediator between overweight and gelotophobia in adolescents? (3) Are gelotophobia and experiences of teasing associated with reduced joy at school, especially in social contexts (i.e., in the schoolyard)?

## Study I

In order to explore the association between gelotophobia, overweight, weight-related teasing and victimization. It is of interest to study whether gelotophobes report physical appearance (including weight-related aspects of appearance) as assumed reasons for being laughed at. In addition, the source of victimization and onset of stigma are of interest. It is hypothesized that striking features in appearance are the predominantly assumed reasons for being laughed at. For gelotophobes, laughter-related experiences in childhood and youth, but not in adulthood, are supposed to be highly relevant. Peers should therefore play the central role as interpersonal source of victimization experiences.

### Method

#### Procedure and Participants

Participants were recruited by Internet contact. Media coverage of feature stories on gelotophobia were utilized to recruit participants by providing a URL that directed interested people to a website. Participants were invited to complete questionnaires on the site. No personal identifying information was taken (for details, see Platt et al., 2012).

Gelotophobia was assessed by the GELOPH<15> (Ruch and Proyer, 2008). The questionnaire for the assessment of gelotophobia is comprised of 15 items (e.g., “When they laugh in my presence I get suspicious.”). Items are positively keyed and responses are given on a four-point Likert scale (1 = strongly disagree; 2 = moderately disagree; 3 = moderately agree; 4 = strongly agree). The GELOPH<15> was used in previous studies and proved to be a reliable and valid instrument for the assessment of gelotophobia (Ruch and Proyer, 2008; Proyer et al., 2009). In the present sample, the GELOPH<15> proved to be reliable, yielding a high internal consistency (Cronbach’s α = 0.91; see Platt et al., 2012).

Of the original English-speaking sample of 622 adults (Platt et al., 2012, Sample I), a sub-sample of 113 participants reported extreme fear of being laughed at (*M* ≥ 3.5; Ruch, 2009; Ruch et al., 2014). Interview data were available for 102 of these participants (63 men, 39 women; mainly from United States, United Kingdom, Australia, India, and Canada; age varied between 18 and 63 years, *M* = 26.70, *SD* = 12.01). Civil status of the participants was distributed as follows: single (*n* = 72), married (*n* = 16), cohabiting (*n* = 11), separated (*n* = 2), and widowed (*n* = 1).

The *Structured Gelotophobia Interview—Written Experimental Form* (Platt and Ruch, 2007, Unpublished; see also Platt et al., 2012) contains a list of 20 questions relating to a variety of issues regarding the onset of the fear of being laughed at, typical ways of dealing with it, thoughts, emotions and actions while being laughed at, bullying experience as well as socio-demographic variables. The interview was administered in a written format. For the present study, only certain aspects of the interview have been analyzed.

#### Data Coding and Analysis

The written responses to the questions submitted online were coded using a stage model of qualitative content analysis (Berg, 2004). After reading the responses for content, primary analytic categories were identified. A coding template was developed based on these responses to establish variables and categories and determine criteria for selection and sorting of content into variables and categories. For the present analysis, only three variables of the interview were analyzed: assumed reason for being laughed at (*assumed reason*), the remembered period of life or age (*onset*), and the interpersonal source of threat (*source*) when the first victimization experience associated with the onset of the fear of being laughed at occurred.

Categories for *assumed reason* were social behavior (e.g., social interaction, having eye contact, having seemed weird to classmates, awkward moments, being over-reactive, grieving for a loved one), physical appearance, tribal [(i.e., ethnic background, religion), residual, and not evident from response; for stigma categories see Goffman, 1963]. The physical appearance category included three subcategories: physical stable and overweight-related (e.g., overweight, fat, obesity, and chubby), physical stable and not overweight-related (e.g., bad skin, big lips, red hair, and being ugly), and physical flexible (e.g., clothes, got glasses). Categories for *onset* (for time period of stigma, see Puhl et al., 2008) were childhood (including early childhood), elementary school, middle school, high school, adulthood, and not evident from response. *Source* categories were peers, family (i.e., brother, siblings, mother, and father), others (teacher, other person), peers and family, peers and others, and not evident from response.

All responses were double coded by the first author and a student research assistant^1^, and inter-rater agreement (Cohen, 1960) was calculated. With all coefficients κ > 0.80, a high agreement between the two coders was achieved.

### Results

Descriptive statistics are presented in **Table 1**. Among the *assumed reasons* for being laughed at the categories social behavior (*n* = 22, 22%), physical appearance (*n* = 18, 18%), and not evident from response (*n* = 58, 57%) were coded most often. The latter category plus tribal (*n* = 1) and residual (*n* = 3) were summarized as the new aggregated category residual (*n* = 62). Among the physical appearance category the sub-category physical stable and overweight-related was reported by six participants (i.e., 33% of the physical appearance category and 6% of all assumed reason categories).

**Table 1:** Variables and categories derived from the Structured Gelotophobia Interview (Study I).

Variable	Description of variable	Categories	Aggregated categories (if applicable)
Assumed reason	Assumed reason for being laughed at		
		Social behavior (*n* = 22)	Social behavior (*n* = 22)
		Physical appearance (*n* = 18)^∗^	Physical appearance (*n* = 18)
		Tribal (*n* = 1)	Residual (*n* = 62)
		Residual (*n* = 3)	
		Not evident from response (*n* = 58)	
Onset	Onset of fear of being laughed at		
		Childhood (*n* = 12) Elementary school (*n* = 38)	Childhood and elementary school (*n* = 50)
		Middle school (*n* = 20) High school (*n* = 9)	Middle school and high school (*n* = 29)
		Adulthood (*n* = 1)	Residual (*n* = 23)
		Not evident from response (*n* = 22)	
Source	Interpersonal source of threat		
		Peers (*n* = 39)	
		Family (*n* = 6)	
		Others (*n* = 1)	
		Peers and family (*n* = 6)	
		Peers and others (*n* = 1)	
		Not evident from response (*n* = 49)	

*N = 102. ^∗^The physical appearance category was comprised of the sub-categories physical stable and overweight-related (n = 6, 33%), physical stable and not overweight-related (n = 9, 50%), and physical flexible (n = 3, 17%).*

*Onset* of the fear of being laughed at had its maximum in elementary school (*n* = 38, 37%) and its minimum in adulthood (*n* = 1, 1%). For the frequencies for the other onset categories see **Table 1**. For further analyses aggregated categories of onset were computed: childhood and elementary school (*n* = 50), middle school and high school (*n* = 29), and residual (*n* = 23) which was comprised of adulthood (*n* = 1) and not evident from response (*n* = 22).

For almost half of the participants (*n* = 49, 48%) interpersonal *source* of victimization behavior was not evident from response. From the remaining responses, the majority of participants reported that their victimization experiences were enacted by peers (*n* = 39, 38% from total sample), followed by family members (*n* = 6, 6%), or a combination of peers and family members (*n* = 6, 6%). All the responses involving peers combined (i.e., peers, peers and family, peers, and others) resulted in total of reported 46 victimization behaviors (45%) related to peers. An aggregated category for source was not computed.

Separate cross tabulations of gender with *assumed reason* and *onset* were performed. χ^2^ tests (*p*s > 0.23) did not show any evidence of an association of gender of participants with the two dimensions.

A cross tabulation of *assumed reason* and *onset* indicated that the distribution of the two interview variables were not independent from each other (see **Table 2**); χ^2^(4, *N* = 102) = 13.95, *p <* 0.01. Whereas social behavior as the assumed reason for being victimized was not related to the onset categories, physical appearance was especially an assumed reason for being victimized during middle school and high school (*n*_o_ = 10, *n*_e_ = 5.1, *z* = 2.8) but not in childhood and elementary school (*n*_o_ = 7, *n*_e_ = 8.8, *z* = 0.9). Results for the residual categories (e.g., overrepresentation of the onset category residual within the assumed reason category residual) will not be interpreted given the heterogeneous nature of both categories.

**Table 2:** Cross tabulation of assumed reason for being laughed at by onset of fear of being laughed at (Study I): observed frequencies and expected frequencies (in parentheses).

	Onset			
Assumed reason	Childhood and elementary school	Middle school and high school	Residual	Total
Social behavior	14 (10.8)	6 (6.3)	2 (5.0)	22
Physical appearance	7 (8.8)	10^∗^ (5.1)	1 (4.1)	18
Residual	29 (30.4)	13^∗^ (17.6)	20^∗^ (14.0)	62
Total	50	29	23	102

*^∗^p < 0.05 (corrected standardized residuals > |1.96|).*

To summarize, findings showed that among extreme gelotophobic individuals social behavior and physical appearance were reported as assumed reasons for being laughed at. For physical appearance this was especially prominent during the age when attending middle or high school.

### Discussion

For individuals with extreme gelotophobia, social behavior and physical appearance were the alleged main reasons for laughter. Weight-related aspects of appearance accounted for one-third of this category. It has also been shown that severe experiences related to the development of gelotophobia for childhood and schooling are reported. Peers seem to be the primary interpersonal source of remembered threat. Therefore, weight-related teasing and mockery by peers in childhood and youth are at least a remarkable aspect in the broader understanding of gelotophobia development.

Findings of the present study are compatible with findings from gelotophobia research. In a sample of 6 to 9-year-olds gelotophobia was positively related with victim status (Proyer et al., 2012). Führ (2010) found that children and adolescents who reported having been a victim of bullying expressed higher levels of the fear of being laughed at. However, these studies did not have a particular focus on weight-related victimization. A study among undergraduate students (Edwards et al., 2010) revealed that higher gelotophobia was associated with memories of being teased about social behavior, performance, academic excellence, and appearance (including the item “I was teased about my weight”). However, the strongest association of gelotophobia was found for teasing related to social behavior.

A limitation of the present study is that in about half of the cases, the interpersonal source of threat and the suspected reason for being laughed at were not apparent from the transcribed interviews. Unfortunately, weight status of participants was also not assessed in the original Structured Gelotophobia Interview. Therefore, a second interview study was conducted to overcome these limitations.

## Study Ii

Aim of the second study was to test whether main findings of Study I can be replicated in a more structured assessment. It was hypothesized that even in a more structured online interview, individuals high in gelotophobia cite aspects of their physical appearance, including weight aspects, in addition to their conspicuous social behavior as assumed reasons for being laughed at. Furthermore, peers are supposed to be the main source of teasing and mockery remembered, with most of the victimization experiences starting in childhood and schooling.

### Method

#### Procedure and Participants

A German-speaking sample of 35 adults (14 men, 21 women) was recruited by Internet contact based on a newspaper article on gelotophobia (“Hoffentlich lacht keiner”, [Hoping nobody is laughing], Frankfurter Allgemeine Sonntagszeitung, published February 19, 2012). Gelotophobia was assessed by the GELOPH<15>(Ruch and Proyer, 2008; Cronbach’s α = 0.83 in the present sample).

Besides demographics (gender, age, and civil status), self-reports of body weight and height were assessed. Of this sample, 63% (*n* = 22) reported at least substantial fear of being laughed at (*M* > 2.5; slight fear [*M* between 2.5 and 3.0], *n* = 13; marked fear [*M* between 3.0 and 3.5, *n* = 8; extreme fear [*M* ≥ 3.5], *n* = 1; for criteria see Ruch, 2009). Data of these 22 participants (10 men, 12 women; 21 Germans, 1 Italian) were used for further analyses. Age varied between 17 and 66 years (*M* = 44.91, *SD* = 14.06). Civil status of the participants was distributed as follows: single (*n* = 7), married (*n* = 11), cohabiting (*n* = 4). Self-reports of weight and height were given by 20 participants (10 men, 10 women). BMI varied between 17.21 and 42.98 (*M* = 25.04, *SD* = 6.02). Distribution of weight categories were as follows: underweight (*n* = 1 woman), normal weight (*n* = 11; 5 men, 6 women), overweight (*n* = 6; 5 men, 1 woman), and obese (*n* = 2 women).

A slightly modified version of the *Structured Gelotophobia Interview—Written Experimental Form* (Platt and Ruch, 2007, Unpublished; see Study I) was administered in a written format with fixed response categories. Questions included the three variables from Study I: assumed reason for being laughed at (*assumed reason*), the remembered period of life or age (*onset*), and the interpersonal source of threat (*source*) when the first victimization experience associated with the onset of the fear of being laughed at occurred. Categories for *assumed reason* (multiple responses possible) were social behavior, physical appearance, mistake, other, and without reason. Categories for *onset* (only one response possible) were early childhood and preschool (age: 0–5 years), elementary school (6–10 years), middle school (11–15 years), high school (16–20 years), and adulthood (sub-categories were 20–30 years, 30–40 years, …, 60–70 years, >70 years). Categories were based on the German school system. *Source* categories (multiple responses possible) were schoolmates, other peers, family members, friends, teachers, and other adults.

### Results

Descriptive statistics are presented in **Table 3**. Among the *assumed reasons* for being laughed at (with multiple responses allowed) the category of social behavior was reported most frequently (*n* = 9, 41%). Physical appearance was mentioned by five individuals (23%, see **Table 3** for the other categories). *Onset* of victimization experiences related to the fear of being laughed at had its maximum in early childhood and preschool (*n* = 10, 45%), followed by elementary school (*n* = 7, 32%) and middle school (*n* = 5, 23%). High school and adulthood were not mentioned. To compare findings for *onset* with those from Study 1, we additionally aggregated categories of *onset*: this yielded *n*s of 17 (77%) for childhood and elementary school, 5 (23%) for middle school and high school, and 0 for adulthood. As interpersonal *sources* of victimization behavior (with multiple responses allowed) participants most often reported schoolmates (*n* = 15, 68%) and other peers (*n* = 9, 41%), family members (*n* = 12, 55%), teachers (*n* = 9, 41%), and other adults (*n* = 7, 32%; see **Table 3** for the other categories).

**Table 3:** Variables and categories derived from the modified Structured Gelotophobia Interview (Study II).

Variable	Description of variable	Categories	Aggregated categories (if applicable)
Assumed reason^∗^	Assumed reason for being laughed at		
		Social behavior (*n* = 9)	
		Physical appearance (*n* = 5)	
		Mistake (*n* = 4)	
		Other (*n* = 6)	
		Without reason (*n* = 8)	
Onset	Onset of fear of being laughed at		
		Early childhood and preschool (*n* = 10) Elementary school (*n* = 7)	Childhood and elementary school (*n* = 17)
		Middle school (*n* = 5) High school (*n* = 0)	Middle school and high school (*n* = 5)
		Adulthood (*n* = 0)	Adulthood (*n* = 0)
Source^∗^	Interpersonal source of threat		
		Schoolmates (*n* = 15)	
		Other peers (*n* = 9)	
		Family (*n* = 12)	
		Friends (*n* = 4)	
		Teachers (*n* = 9)	
		Other adults (*n* = 7)	
		Unknown (*n* = 0)	

*N = 22. ^∗^Multiple responses possible. Therefore, no aggregated categories were computed for this variable.*

In contrast to Study I, the gelotophobia interview variables assumed reason and source were based on a multiple response format. Therefore, associations of these variables with gender and weight status were analyzed separately for each category. Associations of *assumed reason, onset,* and *source* with gender (*N* = 22) and weight status (*N* = 20 due to missing values) were generally low (χ^2^ tests and Fisher’s exact tests, *p*s > 0.10). There was only a significant association between gender and *onset*; Fisher’s exact test, *p* < 0.05. More women than men reported an early onset for the first victimization experience associated with the fear of being laughed at: early childhood and preschool (men: *n* = 2, women: *n* = 8), elementary school or middle school (men: *n* = 8, women: *n* = 4).

Findings showed that for early childhood and the period attending elementary school, first victimization experiences as assumed reasons for the onset of gelotophobia were reported. Schoolmates and other peers as well as family members and teachers were the main *sources* of victimization. Social behavior was more predominant than physical appearance among the most often reported reasons assumed for being laughed at.

### Discussion

In a structured interview, people with gelotophobia also report that they were teased and mocked mainly because of their social behavior but also because of their appearance. These unpleasant experiences began mainly in childhood and during elementary school. In these two points, Study II confirms the results of Study I, although the criterion for the diagnosis of gelotophobia was not as strict as in Study I. However, a striking difference to Study I resulted for the reported source of teasing and harassment. Although in both studies peers and classmates are the main attackers, in Study II family members and teachers were also mentioned remarkably often.

It cannot be ruled out that the greater importance of family members and teachers as sources of teasing in Study I compared to Study II is related to the fact that the samples come from different cultural backgrounds or that the average age of the interviewees in Study II was significantly higher (around 45 years) than in Study I (27 years). Davies (2009) assumes that pressure to conform and maintain harmony and the existence and maintenance of hierarchies are important social variables related to victimization and the development of gelotophobia. The differences in the role of family members and teachers between the two studies may be due to the fact that there may have been a shift from one generation to another in the social factors mentioned by Davies.

Weight status was independent of the variables derived from the interviews. Although almost a quarter of all respondents cited physical appearance as a reason for laughing, the small sample size (22 participants, eight of them were overweight or obese) was far from being ideal for investigating associations between weight status and specific forms of teasing.

To summarize, both interview studies showed that physical appearance was reported by at least some gelotophobic adults as an assumed reason for being laughed at in childhood and adolescence. These preliminary findings, however, cannot support the assumption that the weight status of children and adolescents (i.e., overweight and obesity) may be associated with gelotophobia mediated by weight-related teasing. To test this assumption more directly, Studies III and IV were conducted with students who were not pre-selected according to their degree of gelotophobia.

## Study III

Overweight and obese children and adolescents experience more weight-based teasing and victimization than children and adolescents with normal weight (Puhl and Latner, 2007). Research suggests that weight status is predictive of vulnerability to bullying in peer relationships (Neumark-Sztainer et al., 2002). For example, the prevalence of weight-based teasing by peers was significantly higher among overweight and obese youth (45%) than among normal weight youth (22%; Goldfield et al., 2010). Overweight and obese girls experienced even more weight-related teasing by peers than overweight and obese boys (52% vs. 30%). In addition, teasing about body weight was associated with impaired well-being (anxiety, depression, and negative self-esteem). Among overweight children and adolescents, appearance-related teasing seems to be prevalent, frequent, upsetting, and focusing more on weight than on less stigmatized aspects of appearance (Hayden-Wade et al., 2005).

**FIGURE 1:**
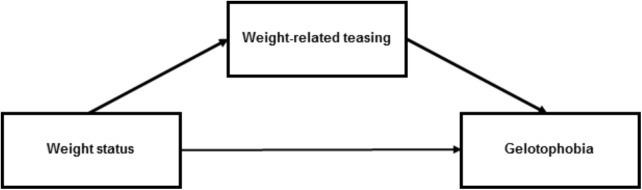
Proposed model: Weight-related teasing as mediator between weight status and gelotophobia.

The present study aimed at analyzing the relationship between weight status, weight-based victimization and gelotophobia among adolescent boys and girls. The main research question is whether weight-related teasing mediates the relationship between weight status (e.g., being overweight or obese) and gelotophobia (see **Figure 1**).

In addition, the association of weight status, teasing and gelotophobia with the adolescents’ body image will be explored. In a representative German sample of 3,254 girls and 3,415 boys aged 11–17 years, a high proportion of normal-weighted girls and boys had a negative overweight-associated body image (i.e., “I think I’m a bit too fat” or “… far too fat”; 49% of the girls compared to 26% of the boys; Kurth and Ellert, 2008), which was associated with reduced well-being. On the other hand, overweight adolescents who perceive their weight about right report a better psychological and physical health than those with realistic self-perceptions (Fuchs et al., 2012). Therefore, not only the association of weight status but also the association of body image with weight-related teasing and gelotophobia is of interest.

### Method

#### Participants and Procedure

Participants were 75 adolescents (boys: *n* = 23, girls: *n* = 52; age: *M* = 13.97 years, *SD* = 1.08 years, Range = 12–16 years), recruited from Swiss middle schools in the Cantons of Zurich and Aargau. Parental and participant consent was required to participate in the study. All data (i.e., demographic information, reports of weight and height, questionnaires) were assessed online (Unipark).

#### Variables and Questionnaires

*Gelotophobia* was assessed by the GELOPH<15>(Ruch and Proyer, 2008; see Study I; Cronbach’s α = 0.87 in the present sample).

##### Teasing

The Teasing Questionnaire-Revised (Strawser et al., 2005; see also Storch et al., 2004) is a 29-item self-report scale designed to measure teasing experiences. Responses are given on five-point Likert-type scale (0 = “I was never teased about this,” 1 = “I was rarely teased about this,” 2 = “I was sometimes teased about this,” 3 = “I was often teased about this,” and 4 = “I was always teased about this”). Domains considered are performance (e.g., “not good at sports”), academic excellence (e.g., “not ‘nerdy”’), social behavior (e.g., “often looked nervous”), family background (e.g., “I had a ‘funny’ name”), and appearance (e.g., “fatter than other kids,” “color or style of hair”). The *teasing (total)* scale showed a good reliability with Cronbach’s α = 0.92 in the present sample.

Based on the literature on the main topics of weight-related teasing (Puhl and Latner, 2007), an additional 6-item subscale on *weight-related teasing* was composed for the present study. One item of the performance factor (“not good at sports”) and five items of the appearance factor (“aspects of my appearance,” “being ugly/unattractive,” “weight,” “way that I dressed,” “fatter than other kids”) were used (Cronbach’s α = 0.88; for similar scales for the assessment of weight-related teasing, see Thompson et al., 1995; Gros et al., 2012).

##### Weight categories

Self-reports of weight and height were assessed to compute BMI scores. Based on age- and gender-specific norms, participants were allocated to weight categories (Kromeyer-Hauschild et al., 2001; see also Cole et al., 2000; Stamm et al., 2010). The original five categories were reduced to three categories by combining the two underweight categories (i.e., extremely underweight: *n* = 4; underweight: *n* = 9) as well as the overweight (*n* = 5) and the obesity category (*n* = 1) in one category each, resulting in the weight categories underweight (*n* = 13; 4 boys, 9 girls), normal weight (*n* = 56; 18 boys, 38 girls) and overweight (*n* = 6; 1 boy, 5 girls).

##### Body image

Participants were asked to evaluate their own weight using a single item: “Do you think you are … (1) far too thin, (2) a bit too thin, (3) just the right weight, (4) a bit too fat, (5) far too fat?” (Kurth and Ellert, 2008). The original five categories were reduced to three categories: 1 (*n* = 2) and 2 (*n* = 14) were combined into “too thin” and 4 (*n* = 30) and 5 (*n* = 0) into “too fat,” resulting in the following distribution of body image: “too thin” (*n* = 16; 5 boys, 11 girls), “just the right weight” (*n* = 29, 9 boys, 20 girls), and “too fat” (*n* = 30, 9 boys, 21 girls).

In addition, dummy-coded variables for weight categories (underweight: yes = 1, no = 0; overweight: yes = 1, no = 0) and body image (“too thin”: yes = 1, no = 0; “too fat”: yes = 1, no = 0) were computed.

#### Data Analysis

The correspondence between weight status and body image was evaluated by Cohen’s kappa (Cohen, 1960). For an initial examination, descriptive statistics and correlations among gelotophobia and teasing and with weight status and body image were computed separately for boys and girls. As a second step, to examine whether there will be an indirect association between overweight and gelotophobia mediated by weight-related teasing, path analyses were computed (Preacher and Hayes, 2004; Hayes, 2013). Mediation analyses were conducted with a SPSS macro using bootstrapping with *z* = 5,000 resamples to compute 95% confidence intervals for the indirect effect.

### Results

Weight categories and body image show a low but significant association (Cohen’s κ = 0.25, *p* < 0.001; 95% confidence interval: 0.08–0.43; see **Table 4**). Separate analyses for male and female adolescents did not result in different associations (κs = 0.25 for boys and girls, resp.). However, among the normal weight group a substantial number of adolescents (23 out of 56, i.e., 41%) perceive themselves as being “too fat.”

**Table 4:** Cross tabulation of weight categories and body image (Study III): observed frequencies.

Weight categories	Body image			
	“Too thin”	“Just right”	“Too fat”	Total
Underweight	**8**	4	1	13
Normal weight	8	**25**	23	56
Overweight	0	0	**6**	6
Total	16	29	30	75

*“Just right” = “just the right weight”. Cells with an expected agreement between weight categories and body image are printed in bold. κ = 0.25, p < 0.001.*

Correlations between gelotophobia, teasing (total) and weight-related teasing as well as the correlations of these variables with weight categories and body image are presented in **Table 5**. Gelotophobia was positively correlated with teasing (total) in girls (*r* = 0.42, *p* < 0.01) and in boys (*r* = 0.41, *p* = 0.06). Weight-related teasing, however, was associated with gelotophobia for girls only (*r* = 0.40, *p* < 0.01). Gelotophobia did not show significant associations with weight categories or body image (e.g., correlation between gelotophobia and underweight in boys: *r* = 0.25, *p* = 0.26; correlation between gelotophobia and overweight in girls: *r* = 0.21, *p* = 0.14). In girls, overweight was significantly associated with teasing (total, *r* = 0.32, *p* < 0.05) as well as weight-related teasing (*r* = 0.41, *p* < 0.01).

**Table 5:** Correlations of gelotophobia and teasing with weight categories and body image and joy at school (Study III).

				Variable		
Variable		*M*	*SD*	Gelotophobia	Teasing (total)	Weight-related teasing
Gelotophobia	Boys	2.06	0.48	1.00		
	Girls	2.19	0.58	1.00		
Teasing (total)	Boys	0.41	0.50	0.41^+^	1.00	
	Girls	0.40	0.40	0.42**	1.00	
Weight-related teasing	Boys	0.49	0.61	0.18	0.78**	1.00
	Girls	0.55	0.79	0.40**	0.82**	1.00
Underweight	Boys	0.17	0.39	0.25	-0.06	-0.22
	Girls	0.17	0.38	-0.11	0.10	-0.02
Overweight	Boys	0.04	0.21	-0.03	-0.12	0.00
	Girls	0.09	0.30	0.21	0.32*	0.41**
Body image “too thin”	Boys	0.22	0.42	-0.10	-0.12	-0.17
	Girls	0.21	0.41	-0.07	-0.09	-0.15
Body image “too fat”	Boys	0.39	0.50	-0.03	-0.33	-0.07
	Girls	0.40	0.50	0.17	0.04	0.24

*Boys: n = 23, girls: n = 52. Gelotophobia and teasing scores are reported as item means (sum scores divided by number of items; Gelotophobia, range: 1–4; Teasing, range: 0–4, Joy, range: 1–4. Weight categories and body image are dummy-coded (1 = yes, 0 = no). ^+^p < 0.06, ^∗^p < 0.05, ^∗∗^p < 0.01 (two-tailed).*

**FIGURE 2:**
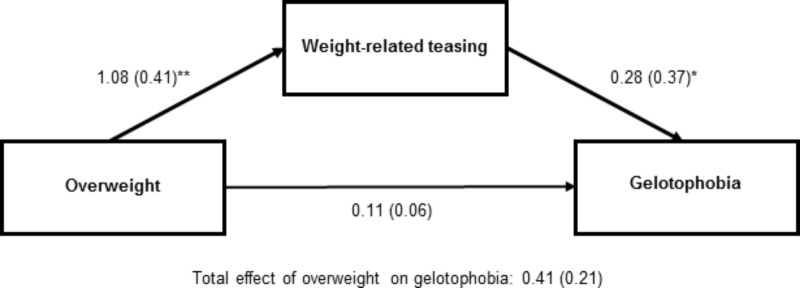
Weight-related teasing as mediator between overweight and gelotophobia in girls (*N* = 52, Study III). Unstandardized regressions coefficients and standardized regression coefficients (in parentheses) are based on item mean scores for weight-related teasing and gelotophobia, respectively. Overweight categories: 0 = no, 1 = yes. ^∗^*p* < 0.05, ^∗∗^*p* < 0.01.

In the tested mediation model the independent variable was overweight (dummy-coded), mediator was weight-related teasing, and the dependent variable was gelotophobia. Only among girls the indirect effect was significant. The 95% CI obtained for the indirect effect of overweight status on gelotophobia by bootstrapping was 0.23 (CFI: 0.03 to 0.63) and did not include 0 (*z* = 5,000 bootstrap resamples), indicating an indirect-only mediation (Zhao et al., 2010) since the direct path was not significant^2^. The results for the interplay between overweight status, weight-related teasing, and gelotophobia in girls are shown in **Figure 2**. Weight-related teasing was higher in overweight than in non-overweight adolescent girls. Furthermore, gelotophobia was higher with enhanced weight-related teasing. Overweight status was not directly related to gelotophobia. However, overweight status and gelotophobia were indirectly associated by weight-related teasing.

### Discussion

Weight status and body image showed a significant but small association. Especially the high proportion of normal weight adolescents with an unrealistic overweight-related body image was in accordance with a previous study by Kurth and Ellert (2008). In some contrast to their study, however, is the finding that in the present study body image was unrelated to gelotophobia. According to Kurth and Ellert (2008) body image seems to be crucial for psychological well-being. Given the conception of gelotophobia as a shame-bound anxiety, this could have been expected. However, gelotophobia cannot be fully explained by negative affect and is also sufficiently different from social anxiety (Ruch et al., 2014). To further explore the association between body image and gelotophobia, a larger sample size seems to be needed.

Findings of the present study give some support for the assumption that weight-related teasing may play a role in gelotophobia, at least in girls. Overweight adolescent girls reported increased weight-related teasing which itself was associated with gelotophobia. A similar association could not be observed in boys. The strong association between weight status and weight-related teasing in girls is in accordance with the findings by Goldfield et al. (2010). However, they found an (albeit smaller) association for boys as well. In general, the significant path between weight-related teasing and gelotophobia in girls parallels findings on memories of appearance-related teasing in gelotophobic adults (Studies I and II) and adolescents (Edwards et al., 2010). In these studies, however, findings were independent from gender or gender differences were not explicitly tested. The fact that no hypothesis-compliant correlations could be demonstrated for boys in the present study must also be viewed critically with regard to the sample examined. The proportion of boys was not only low overall, but only one boy was overweight. To overcome the limitations of the study a replication of the performed analyses based on a larger sample size is needed.

## Study Iv

Main objective of the study was to further examine the relationship between weight status, weight-based victimization and gelotophobia among adolescent boys and girls. Based on a larger sample, the hypothesis to be tested is whether weight-related teasing mediates the relationship between weight status (e.g., being overweight or obese) and gelotophobia. Again, correlations of weight status, teasing, and gelotophobia with the adolescents’ body image will be analyzed.

Previous research has repeatedly demonstrated that weight-related teasing and victimization in the school setting is associated with negative affect in adolescent boys and girls (for example, see Puhl and Luedicke, 2012). Given the importance of positive emotions at school (for an overview, see Pekrun et al., 2018), however, the present study will broaden the perspective by adding measures of joy for two prototypical school situations (i.e., writing a class test, being together with others in the school yard; Eschenbeck et al., unpublished). It is hypothesized that especially joy in the social situation (i.e., at the schoolyard) shows a strong negative association with teasing and gelotophobia.

### Method

#### Participants and Procedure

Participants were recruited from secondary schools in southern Germany. Complete data sets were available for 178 adolescents (boys: *n* = 93, girls: *n* = 85; age: *M* = 13.80 years, *SD* = 1.13, Range = 12–16 years). Parental consent was required to participate in the study. Participants and their parents provided their informed consent prior to the start of the study. Adolescents completed a self-report questionnaire in their classes. The measures were administered by trained students.

#### Variables

*Gelotophobia* was assessed by the GELOPH<15>(Ruch and Proyer, 2008; see Study I), with Cronbach’s α = 0.87 in the present study.

##### Teasing

The Teasing Questionnaire-Revised (Strawser et al., 2005; see Study III) was applied, yielding in a score for teasing (total) with α = 0.93 and a score for weight-related teasing with α = 0.86 (for scale description see Study III).

*Joy at school* was assessed by two subscales of the Multidimensional Anxiety Inventory for Children and Adolescents (MAICA; Eschenbeck et al., unpublished). Two school-related scenarios were presented: *class test* (“Imagine you are taking a test at school”) and *schoolyard* (“Imagine you are together with your class mates [e.g., in the schoolyard, in the classroom during your break, in the locker room, on the way to school]”). Participants rated their emotions on a four-point Likert scale (almost never/never = 1, sometimes = 2, often = 3, almost always/always = 4) on five items (e.g., “I feel good,” “I’m cheerful”) for each of the two school scenarios presented. Reliabilities (Cronbach’s α) were α = 0.89 for joy during school test and α = 0.87 for joy at the schoolyard.

##### Weight status

Participants reported height and weight. BMI was calculated from both self-reported height and weight (BMI = weight in kilograms/height in meter^2^). Overweight and obesity were defined using the BMI reference values of Kromeyer-Hauschild et al. (2001) according to the recommendations of the German Working Committee on Obesity in Children and Adolescents. As in Study III, the original five categories were reduced to three categories by combining the two underweight categories (i.e., extremely underweight: *n* = 10; underweight: *n* = 12) as well as the overweight (*n* = 7) and the obesity category (*n* = 5) in one category each, resulting in the weight categories underweight (*n* = 22; 8 boys, 14 girls), normal weight (*n* = 144; 76 boys, 68 girls) and overweight (*n* = 12; 9 boys, 3 girls).

*Body image* was assessed by single item: “Do you think you are … (1) far too thin, (2) a bit too thin, (3) just the right weight, (4) a bit too fat, (5) far too fat?” (Kurth and Ellert, 2008; see Study III). As in Study III, the original five categories were reduced to three categories: “too thin” (*n* = 21; “far too thin”: *n* = 4; “a bit too thin”: *n* = 17), “just the right weight” (*n* = 96), and “too fat” (*n* = 61; “a bit too fat”: *n* = 54; “far too fat”: *n* = 7).

In addition, dummy-coded variables for weight categories (underweight: yes = 1, no = 0; overweight: yes = 1, no = 0) and body image (“too thin”: yes = 1, no = 0; “too fat”: yes = 1, no = 0) were computed.

#### Data Analysis

Data analysis was similar to Study III. Agreement between weight status and body image was evaluated by Cohen’s kappa. Descriptive statistics and correlations among gelotophobia and teasing and with weight status, body image and joy at school were computed. Again, the analyses were performed separately for boys and girls. In addition, 2 × 3 ANOVAs with the between-subject factors gender and weight status (underweight, normal weight, overweight) or body image (“too thin,” “just right,” “too fat”) and the dependent variables gelotophobia and teasing were calculated. To examine whether there will be an indirect association between weight status and gelotophobia mediated by weight-related teasing, path analyses were computed as described for Study III.

### Results

Weight categories and body image show a low but significant association (Cohen’s κ = 0.22, *p* < 0.001; 95% confidence interval: 0.08–0.36; see **Table 6**). Separate analyses for male and female adolescents yielded a substantial correspondence between weight category and body image for boys (κ = 0.44, *p* < 0.001; 95% confidence interval: 0.22–0.65) and a much smaller one for girls (κ = 0.08, *p* = 0.07; 95% confidence interval: 0.00–0.24). The difference between boys and girls was mainly a result of a high proportion of girls with normal weight reporting a body image of “too fat” (54%), whereas in boys this proportion was lower (14%). Correspondingly, among the underweight girls the percentage of those with a body image of “just right” or “too fat” was 64%, whereas this proportion was 38% in boys.

**Table 6:** Cross tabulation of weight categories and body image (Study IV): observed frequencies.

Weight categories	Body image			
	“Too thin”	“Just right”	“Too fat”	Total
Underweight	**10**	9	3	22
Normal weight	11	**85**	48	144
Overweight	0	2	**10**	12
Total	21	96	61	75

*“Just right” = “just the right weight.” Cells with an expected agreement between weight categories and body image are printed in bold. κ = 0.22, p < 0.001.*

Correlations between gelotophobia and teasing as well as the correlations of these variables with weight categories, body image and joy are presented in **Table 7**. For both boys and girls, gelotophobia was correlated positively with teasing (total) and weight-related teasing and negatively with joy at the schoolyard. The negative association between gelotophobia and joy at school was more pronounced among girls (*r* = -0.50) than among boys (*r* = -0.20; *z* = 2.27, *p* < 0.05). With regard to associations of gelotophobia with weight status and body image, only a significant correlation between gelotophobia and underweight status in boys emerged. Underweight boys reported higher fear auf being laughed at as non-underweight boys.

**Table 7:** Correlations of gelotophobia and teasing with weight categories, body image and joy at school (Study IV).

				Variable		
Variable		*M*	*SD*	Gelotophobia	Teasing (total)	Weight-related teasing
Gelotophobia	Boys	1.57	0.47	1.00		
	Girls	1.89	0.55	1.00		
Teasing (total)	Boys	0.25	0.31	0.52^∗∗^	1.00	
	Girls	0.33	0.40	0.62^∗∗^	1.00	
Weight-related teasing	Boys	0.28	0.44	0.49^∗∗^	0.75^∗∗^	1.00
	Girls	0.46	0.75	0.53^∗∗^	0.90^∗∗^	1.00
Underweight	Boys	0.09	0.28	0.30^∗∗^	0.38^∗∗^	0.32^∗∗^
	Girls	0.16	0.37	-0.07	-0.18	-0.18
Overweight	Boys	0.10	0.30	0.01	-0.07	0.14
	Girls	0.04	0.19	-0.07	0.02	0.11
Body image “too thin”	Boys	0.11	0.31	0.17	0.36^∗∗^	0.28^∗^
	Girls	0.13	0.34	0.00	0.13	-0.14
Body image “too fat”	Boys	0.19	0.40	0.05	-0.12	0.08
	Girls	0.51	0.50	0.14	0.21	0.27^∗^
Joy (class test)	Boys	2.14	0.79	0.01	-0.21^∗^	-0.12
	Girls	1.79	0.67	-0.04	-0.08	-0.13
Joy (schoolyard)	Boys	3.25	0.72	-0.20^∗^	-0.22^∗^	-0.26^∗^
	Girls	3.22	0.79	-0.50^∗∗^	-0.62^∗∗^	-0.61^∗∗^

*Boys: n = 93, girls: n = 85. Gelotophobia, teasing, and joy scores are reported as item means (sum scores divided by number of items; Gelotophobia, range: 1–4; Teasing, range: 0–4, Joy, range: 1–4. Weight categories and body image are dummy-coded (1 = yes, 0 = no). ^∗^p < 0.05, ^∗∗^p < 0.01 (two-tailed).*

In general, results for teasing are similar to those reported for gelotophobia. Both teasing (total) and weight-related teasing were elevated in underweight boys. Also, joy at school was correlated with both teasing measures, whereas again the correlations were stronger for girls than for boys (*z*s > 3.00, *p*s < 0.01). A significant negative correlation for joy at class test with teasing (total) emerged but only for boys.

Associations of body image with weight-related teasing varied as a function of gender. For boys, a body image of seeing oneself as “too thin” was associated with weight-related teasing as well as with teasing (total). In contrast, for girls, a body image of seeing oneself as “too fat” that was associated with weight-related teasing.

**FIGURE 3:**
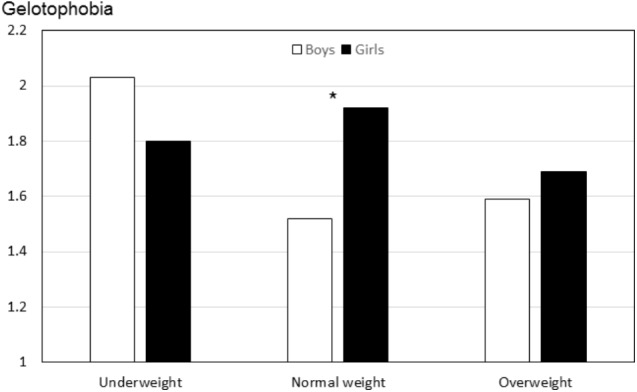
Gelotophobia as a function of gender and weight categories (Study IV). Underweight (boys: *n* = 8, girls: *n* = 14), Normal weight (boys: *n* = 76, girls: *n* = 68), Overweight (boys: *n* = 9, girls: *n* = 3). Significant gender differences within weight groups are marked. ^∗^*p* < 0.05.

For a simultaneous analysis of either gender and weight status or gender and body image 2 × 3 ANOVAs with the dependent variables gelotophobia and teasing were calculated. The three 2 × 3 ANOVAs with gender and weight status (underweight, normal weight, overweight) all resulted in significant interactions of gender by weight status for gelotophobia; *F*(2,172) = 3.55, *p* < 0.05; and for teasing (total); *F*(2,172) = 7.02, *p* < 0.01; and for weight-related teasing; *F*(2,172) = 4.94, *p* < 0.01. **Figure 3** illustrates the findings for gelotophobia. Underweight boys reported the highest gelotophobia among boys whereas both normal weight and overweight girls reported the highest gelotophobia among girls. Probably due to low number of underweight and overweight participants, however, only for the normal weight groups gender differences were statistically significant.

**FIGURE 4:**
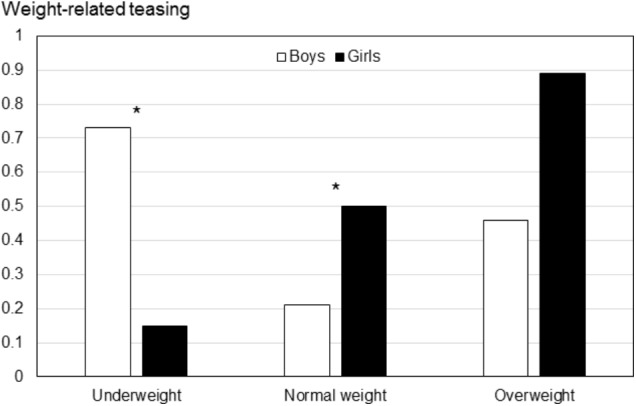
Weight-related teasing as a function of gender and weight categories (Study IV). Underweight (boys: *n* = 8, girls: *n* = 14), Normal weight (boys: *n* = 76, girls: *n* = 68), Overweight (boys: *n* = 9, girls: *n* = 3). Significant gender differences within weight groups are marked. ^∗^*p* < 0.05.

Both analyses for the teasing variables indicated that underweight boys (teasing [total]: *M* = 0.73; weight-related teasing: *M* = 0.63) and overweight girls (teasing [total]: *M* = 0.36; weight-related teasing: *M* = 0.89) report the highest amount of teasing compared to almost all other groups (teasing [total]: *M*s between 0.16 and 0.36; weight-related teasing: *M*s between 0.15 and 0.50). Only for girls, teasing (total) in both overweight and normal weight girls was the same (*M*s = 0.36). **Figure 4** illustrates the findings for weight-related teasing as a function of gender and weight category.

The 2 × 3 ANOVAs with gender and body image (“too thin,” “just the right weight,” “too fat” resulted in significant gender main effect for gelotophobia with higher scores for girls than for boys; *F*(1,172) = 5.86, *p* < 0.05 (see **Table 7**)^3^.

**FIGURE 5:**
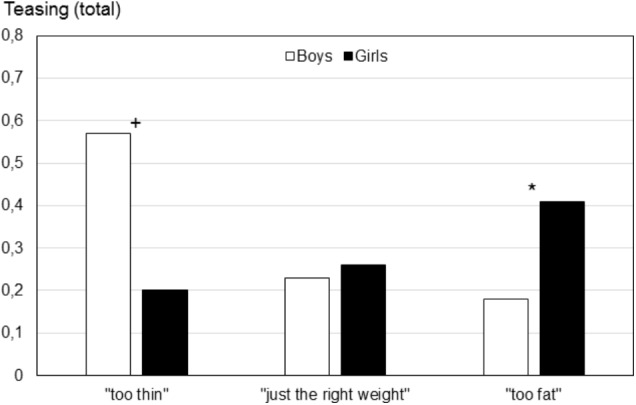
Teasing (total) as a function of gender and body image (Study IV). “too thin“ (boys: *n* = 10, girls: *n* = 11), “just the right weight“ (boys: *n* = 65, girls: *n* = 31), “too fat“ (boys: *n* = 18, girls: *n* = 43). Significant gender differences within body image groups are marked. ^∗^*p* < 0.05, ^+^*p* < 0.07.

**FIGURE 6:**
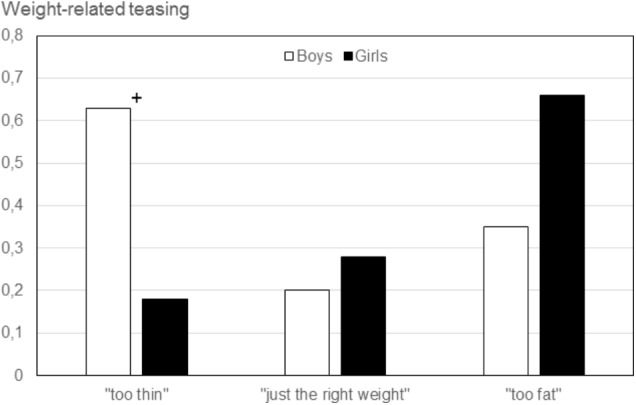
Weight-related teasing as a function of gender and body image (Study IV). “too thin“ (boys: *n* = 10, girls: *n* = 11), “just the right weight“ (boys: *n* = 65, girls: *n* = 31), “too fat“ (boys: *n* = 18, girls: *n* = 43). Significant gender differences within body image groups are marked. ^+^*p* < 0.06.

For the two teasing variables interactions of gender by body image were significant. Interactions for teasing (total), *F*(2,172) = 5.61, *p* < 0.005, as well as weight-related teasing, *F*(2,172) = 3.08, *p* < 0.05, are shown in **Figures 5**, **6**, resp. For both teasing (total) and weight-related teasing, boys who perceived themselves as “too thin” and girls who perceived themselves as “too fat” showed the highest scores.

**FIGURE 7:**
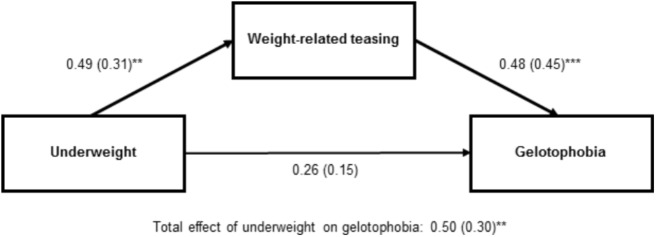
Weight-related teasing as mediator between underweight and gelotophobia in adolescent boys (*n* = 93, Study IV). Unstandardized regressions coefficients and standardized regression coefficients (in parentheses) are based on item mean scores for weight-related teasing and gelotophobia, respectively. Underweight categories: 0 = no, 1 = yes. ^∗∗^*p* < 0.01, ^∗∗∗^*p* < 0.001.

Finally, we examined whether there was an indirect association between weight status and gelotophobia mediated by weight-related teasing. Path analyses were computed separately for boy boys and girls, each with either overweight or underweight (dummy-coded) as the predictor. Only for boys a significant mediation effect was obtained. The independent variable was underweight (dummy-coded), mediator was teasing (total), and the dependent variable was gelotophobia. The 95% CI obtained for the indirect effect of underweight status on gelotophobia by bootstrapping was 0.24 (CFI: 0.04–0.64) and did not include 0 (*z* = 5,000 bootstrap resamples). The results for the interplay between underweight status, weight-related teasing and gelotophobia are shown in **Figure 7**. The direct effect of being underweight on gelotophobia was fully mediated by weight-related teasing^4^. The findings indicate that gelotophobia in underweight adolescent boys is mainly a function of their experience of being teased.

### Discussion

Findings of this study in part replicate the findings of Study III. First of all, the positive association between weight-related teasing and gelotophobia could be replicated for girls and extended to boys. Therefore, findings are in accordance with previous research on appearance-related teasing and gelotophobia (Edwards et al., 2010) and on weight-related teasing and psychological well-being (Goldfield et al., 2010; Puhl and Luedicke, 2012; Zuba and Warschburger, 2017).

Once again, weight status and body image were significantly but weakly associated. Above all, the high proportion of normal-weight participants with an unrealistically overweight body image, which was particularly pronounced in the present study among girls, corresponded to the previous findings (Kurth and Ellert, 2008).

Concerning the association of weight status with gelotophobia and the mediation effect for weight-related teasing, a comparison of the present study with Study III becomes more difficult. A mediation effect emerged again. In the present study, underweight contributed to gelotophobia via weight-related teasing in boys only. In Study III, however, overweight predicted gelotophobia via weight-related teasing in girls only. The rather low number of boys in Study III and the low number of overweight girls in Study IV make it difficult to directly compare the findings of the two studies. Accepting these limitations, however, the findings of these first studies on the joint analysis of weight status, weight-related teasing and gelotophobia point to an association between weight status and gelotophobia mediated by weight-related teasing, albeit gender differences seem to play a crucial role. This was obvious also for the findings on body image.

Girls who perceived themselves as “too fat” as well as boys who perceived themselves as “too thin” reported the highest teasing scores. According to a review by Cohane and Pope (2001), although boys generally display less overall body concern than girls, many boys of all ages report dissatisfaction with their bodies, often associated with reduced self-esteem. Whereas girls typically wanted to be thinner, boys frequently wanted to be bigger. However, most studies failed to distinguish between “bigness” due to increased muscle and that due to fat. The male muscular body type seems to represent the dominant cultural ideal (Wienke, 1998). Assessment of body image in boys and men, therefore, should not only rely on fat but also on muscularity, resulting in two-dimensional assessment approaches (Cafri and Thompson, 2004). A recent study with male and female adolescents aged 12–16 years (Hoffmann and Warschburger, 2017) revealed more pronounced weight and shape concern in females than males and more pronounced muscularity concern in males than females.

For both teasing (total) and weight-related teasing, negative correlations with joy at school emerged. This finding is in accordance with findings from a study involving more than 90,000 14–16 year old Finish adolescents (Konu et al., 2002). Not being victimized at school was an important social relationship variable associated with psychological health and well-being.

Gelotophobia was related to reduced joy at the schoolyard but not during class test. Although the significant correlations were stronger in girls than in boys, the pattern of findings for the public situation (i.e., at the schoolyard) and the more private performance situation (class test) support the construct validity of the gelotophobia concept. According to Titze (2009) gelotophobic individuals lack liveliness, spontaneity, and joy (see also Platt and Forabosco, 2012). According to Ruch et al. (2014) fear of being laughed cannot simply be reduced to a facet of anxiety, negative affectivity, or neuroticism. The social context is crucial as well. Emotions (either joy or anxiety) when writing a class test seem to be unrelated to gelotophobia because in this situation there is no risk of becoming a victim of teasing or ridicule. Being together with others at the schoolyard, however, may be extremely stressful for gelotophobic students, especially if one takes into account that the behavior on the schoolyard is less standardized than during a class test and that a possible intervening teacher is not nearby.

## General Discussion

The present series of four studies is the first attempt to apply the findings on the connections between obesity, victimization, and well-being (e.g., Puhl and Latner, 2007; Lampard et al., 2014) to the research on teasing and gelotophobia (Edwards et al., 2010; Ruch et al., 2010). It was investigated whether overweight, mediated by weight-related teasing, was also related to gelotophobia. The general discussion will focus on this assumed association pattern.

The two interview studies with adults with pronounced gelotophobia (Studies I and II) indicated that in childhood and adolescence the external appearance (including weight-related aspects) was seen as a possible cause (in addition to social behavior) for teasing and ridicule related to gelotophobia development.

The two correlational studies with adolescents (Studies III and IV) principally confirmed the connection between teasing and gelotophobia reported in the literature (e.g., Edwards et al., 2010). In addition, however, there were also first indications that weight status, mediated by weight-related teasing, may be associated with gelotophobia. Overweight status in girls (in Study III) and underweight status in boys (in Study IV) was related to weight-related teasing, which in turn was accompanied by increased gelotophobia. The reported mediation effect for girls from Study III could not be replicated in Study IV. Similarly, the mediation effect found for boys found in Study IV could not be observed in Study III. This may be due to the limitations within both samples already discussed within these studies.

Especially the sample sizes in these studies can be viewed as critical. Therefore, for the pronounced finding in Study IV of weight-related teasing as mediator between underweight and gelotophobia in adolescent boys, *post hoc* simulation studies were performed. Two Monte Carlo simulations run for the mediation model in two artificial data sets and 10,000 iterations revealed that power was only 0.62 for the direct effect of underweight on gelotophobia (power of the other regressions was sufficient, i.e., 0.80). Monte Carlo simulations with 10,000 iterations replicated in two artificial data sets showed that *N* = 151 boys yielded sufficient power for the regressions involved in the proposed mediation model.^5^ Further research with more statistical power is warranted to investigate the role of teasing on the relationship between weight status and gelotophobia in more detail.

The findings of this set of preliminarily studies on appearance, body weight, weight-related teasing and gelotophobia make it worthwhile to further investigate the role of weight-related teasing for gelotophobia in male and female children, adolescents, and adults. However, several major improvements and extensions could then be made: (1) As already stated above, larger samples of underweight and overweight boys and girls would have to be examined. This would not only result in more robust findings but also allow to include measures of coping (e.g., social support) as potential buffers in the mediation models (Reiter-Purtill et al., 2017). (2) It would be advisable to objectively record the weight status. (3) Multi-dimensional assessment procedures could be used to capture the body image, taking into account not only weight perceptions but also figure and muscularity. (4) If all these points were implemented in a longitudinal design with children and adolescents, important further insights into the role of body weight and body image as well as teasing and victimization for mental well-being and especially gelotophobia could be expected. Recent research by Zuba and Warschburger (2017) suggests that the experience of weight teasing and internalization of weight bias is more important than weight status in explaining psychological functioning among children and indicate a need for appropriate prevention and intervention approaches. Further knowledge on the fear of being laughed at may contribute to deal with this challenge.

## Ethics Statement

Studies I, II, and III were conducted following the ethical guidance of the University of Zurich ethics checklist. Full disclosure and informed consent was provided prior to participation in the study by clicking on an accept and continue link on the website. No participant had access to the study without agreeing. Study IV was conducted according to the ethical guidelines of the German Psychological Society. Study IV was approved by the state education authority. In Studies III and IV, children and their parents gave their informed consent prior to the start of the study.

## Author Contributions

All listed authors contributed meaningfully to the paper. TP and WR developed the concept of Study I. C-WK, TP, and WR developed the concept of Studies II and III. C-WK, HE, MH, and UH-D developed the concept and design of Study IV. C-WK, TP, and WR contributed to the design of Studies I, II, and III. C-WK, TP, and WR analyzed and interpreted the data of Studies I, II, and III. C-WK, HE, UH-D, and MH analyzed and interpreted the data of Study IV. C-WK prepared the draft manuscript, and HE, UH-D, MH, TP, and WR provided critical revisions. All authors approved the final version to be published, and agree to be accountable for all aspects of the work in ensuring that questions related to the accuracy or integrity of any part of the work are appropriately investigated and resolved.

## Conflict of Interest Statement

The authors declare that the research was conducted in the absence of any commercial or financial relationships that could be construed as a potential conflict of interest.

## Footnotes

1 We would like to thank Andreas Vuori for his valuable support in coding the *Structured Gelotophobia Interview—Written Experimental Form* in Study I.

2 Similar findings resulted when the total sample (*N* = 75) with both boys and girls or teasing (total) as the mediator were analyzed.

3 In the ANOVAs of gender by weight status the main effect of gender on gelotophobia did not reach significance.

4 Similar findings resulted when teasing (total) as the mediator was analyzed. The 95% CI obtained for the indirect effect of underweight status on gelotophobia by bootstrapping was 0.30 (CFI: 0.02 to 0.87).

5 To estimate *post hoc* the necessary *N* to reach a power of 0.80 for each regression, we produced 500 simulations with *N* = 20, 500 simulations with *N* = 30, 500 simulations with *N* = 40 … and 500 simulations with *N* = 300 (Beaujean, 2014). We did this simulation with two different seeds (565 and 123). Both simulations showed approximately the same result for each factor of interest. The power analysis was done with R. For fitting the mediation for the simulation we used the lavaan package (Rosseel, 2012) and for running the Monte Carlo simulation we used the simsem package (Pornprasertmanit et al., 2016).
